# Alternative splicing of a cryptic exon embedded in intron 6 of *SMN1* and *SMN2*

**DOI:** 10.1038/hgv.2016.40

**Published:** 2016-12-01

**Authors:** Satomi Yoshimoto, Nur Imma Fatimah Harahap, Yuko Hamamura, Mawaddah Ar Rochmah, Ai Shima, Naoya Morisada, Masakazu Shinohara, Toshio Saito, Kayoko Saito, Poh San Lai, Masafumi Matsuo, Hiroyuki Awano, Ichiro Morioka, Kazumoto Iijima, Hisahide Nishio

**Affiliations:** 1 Department of Community Medicine and Social Healthcare Science, Kobe University Graduate School of Medicine, Kobe, Japan; 2 Department of Pediatrics, Kobe University Graduate School of Medicine, Kobe, Japan; 3 Department of Neurology, Toneyama National Hospital, Osaka, Japan; 4 Institute of Medical Genetics, Tokyo Women’s Medical University, Tokyo, Japan; 5 Department of Paediatrics, Yong Loo Lin School of Medicine, National University of Singapore, Singapore, Singapore; 6 Department of Medical Rehabilitation, Faculty of Rehabilitation, Kobe Gakuin University, Kobe, Japan

## Abstract

Both survival of motor neuron (*SMN*) genes are associated with spinal muscular atrophy; mutations in *SMN1* cause the disease, and *SMN2* modulates its severity. It is established that different alternative splicing of exon 7 occurs for *SMN1* and *SMN2*, and a cryptic exon was recently found in intron 6 of both genes. Here, we characterize this cryptic exon and clarify its alternative splicing pattern in control and spinal muscular atrophy cells.

The survival of motor neuron (*SMN*) genes, *SMN1* and *SMN2*, are closely related to the development and severity of spinal muscular atrophy (SMA).^1^ Mutations in *SMN1*, but not in *SMN2*, are recognized as causing SMA because *SMN1* is completely deleted in more than 95% of SMA patients and is deleteriously mutated in the remaining patients.^2^ *SMN2* is now considered to be a modifying factor of the SMA phenotype.^3,4^ Without exception, all SMA patients retain *SMN2.*^2^

Different patterns of alternative splicing have been observed for *SMN1* and *SMN2.*^1^ *SMN1* exclusively produces a full-length (FL) functional *SMN1* transcript. In contrast, less than 10% of *SMN2* transcripts are FL, and more than 90% lack exon 7 (Δ7).^5^ In SMA, the low levels of FL-SMN protein generated from *SMN2* may partially compensate for the lack of *SMN1*. Progress in our understanding of *SMN* splicing mechanisms has spurred the development of therapeutic compounds that modify the splicing of *SMN2* exon 7 to increase levels of FL-SMN protein, and several SMA clinical trials with such therapeutic compounds, including ISIS-SMNRx (nusinersen) and RG7800, are currently in progress.^6^

However, new alternatively spliced products with a cryptic exon in intron 6 of both *SMN1* and *SMN2* have recently been registered in the Ensemble database: ENST00000506239.6 is an *SMN1* transcript, and ENST00000506734.5 is an *SMN2* transcript (the data for *SMN1* are also registered as NCBI Reference Sequence XP_011541898.1). Because the cryptic exon is located upstream of exon 7, we named this exon ‘exon 7a’. Although splicing of exon 7 in the *SMN* genes has been thoroughly studied, the details of exon 7a splicing have not yet been determined. In this study, we characterized the cryptic *SMN* exon, exon 7a, and clarified its alternative splicing pattern in control and SMA fibroblast cells. Control fibroblasts were grown from the skin of a 30-year-old healthy man who carried two copies of each of *SMN1* and *SMN2*, and SMA fibroblasts were grown from the skin of a 13-year-old SMA female who carried three copies of *SMN2* and no copy of *SMN1*.^7^ In addition, control white blood cells were collected from a 21-year-old healthy female who carried two copies of *SMN1* and no copy of *SMN2*. This study was approved by the Ethical Committee of Kobe University, and informed consent was obtained from the patient and her parents as well as the two healthy individuals.

First, to confirm the presence of an alternatively spliced *SMN* product with exon 7a, we performed reverse transcription–PCR (RT–PCR) analysis using primers to amplify exon 6 and exon 7a (Ex6-F: 5′-CTCCCATATGTCCAGATTCTCTTG-3′ and Ex 7a-R: 5′-CCCAGATCTTTGTGCATTAA-3′; Figure 1a). We obtained an amplified product from the control fibroblast cells (‘Mock’ in Figure 1c) and SMA fibroblast cells (data not shown), and DNA sequencing revealed that it corresponds to a transcript containing exon 6-exon 7a. This observation confirmed the presence of an alternative transcript with exon 7a in both the control and SMA fibroblasts. However, the exon 7a-containing *SMN* transcript level was too low to be detectable by RT–PCR using primers for exon 6 and exon 8 (data not shown). Thus, exon 7a-specific primers were necessary to detect the exon 7a-containing *SMN* transcript by RT–PCR.

Next, to determine the alternative splicing pattern of exon 7a, we performed fluorescent RT–PCR using exon 7a and exon 8 primers (Ex 7a-F: 5′-(FAM)-ACAGGGTTTCACTGTGTTAGCC-3′ and Ex-8R: 5′-ACTGCCTCACCACCGTGCTGG-3′) (Figure 2a). The major splicing product containing exon 7a was 'exon 6-exon 7a-exon 7-exon 8' in both control and SMA fibroblast cells (Figure 2b). However, the transcript containing 'exon 6-exon 7a-exon 8', which is described in the Ensemble database, was only a minor splicing product in our study. Interestingly, the 'exon 6-exon 7a-exon 8' transcript was not detected in white blood cells from the control individual retaining *SMN1* but lacking *SMN2* (Figure 2b). Although it is not certain if such a phenomenon occurs in every tissue, this observation suggests that the 'exon 6-exon 7a-exon 8' transcript is usually generated by *SMN2* but not by *SMN1* (Figure 2c).

We also determined the entire nucleotide sequence of exon 7a (Figure 1b). When the nucleotide sequence was examined within the context of the open reading frame of the *SMN* transcript, two premature termination codons, TGA and TAA, were identified at 58 and 17 bp upstream of exon 7a, respectively. Thus, the exon 7a-containing *SMN* transcript is a possible target of nonsense-mediated decay (NMD) because it follows the ‘50–55 nt rule’ of NMD.^8^

Cycloheximide (CHX) is an NMD inhibitor.^9^ To examine whether the exon 7a-containing *SMN* transcript is an NMD target, we compared CHX (100 μg/ml)-treated and non-treated (mock) control fibroblasts. The *SMN* transcript with exon 6 and 7a and a reference gene transcript (*GAPDH*) were amplified; the primers used to amplify *GAPDH* were 5′-GAGTCAACGGATTTGGTCGT-3′ and 5′-GACAAGCTTCCCGTTCTCAG-3′. The relative expression level of the exon 7a-containing *SMN* transcript in CHX-treated cells was four times higher than that in non-treated cells (Figure 1c). This finding strongly suggests that the exon 7a-containing *SMN* transcript is a target of NMD.

Further studies are necessary to clarify the biological function of the exon 7a-containing *SMN* transcript. As alternative splicing is a mechanism for the quantitative control of gene expression, which involves the use of NMD,^10^ the exon 7a-containing *SMN* transcript may have a role in attenuating the function of the FL-*SMN* transcript or the FL-SMN protein. If this is the case, when SMA patients are treated with a splicing-modulation therapy, determining the expression level of the exon 7a-containing *SMN* transcript may be essential to avoid unexpected off-target effects of the therapeutic compounds.

In conclusion, we characterized *SMN* exon 7a and its splicing characteristics. *SMN* exon 7a has two premature termination codons and is a target of NMD. Further studies are needed to clarify whether alternative splicing involving exon 7a regulates *SMN* gene expression.

## Note added in proof

During the submission of our manuscript, a study performed by Seo *et al.*^11^ was published online. The authors report the same transcript in intron 6 and termed this 109 bp sequence ‘exon 6B’. They confirmed our findings that exon 7a is derived from both *SMN1* and *SMN2* genes. In addition, they showed that the exon 6B-containing *SMN* transcript is the target of NMD, which is consistent with our results for exon 7a.

In our experiment using conventional PCR, the exon 7a-containing *SMN2* transcript could not be amplified without the use of specifically targeted primers, suggesting that its expression level was much lower than that of the Δ7- and FL-SMN2 transcripts. Using a ^32^P-labeled multi-exon skipping detection assay (MESDA), Seo *et al.*^11^ also demonstrated that the expression level of the exon 6B-containing *SMN2* transcript was lower than that of the Δ7- and FL-*SMN2* transcripts.

Seo *et al.*^11^ investigated the stability, function and localization of the SMN 6B protein, which contains exon 6B, through overexpression of *SMN* cDNA plasmid constructs with a 3XFLAG-tag anti-FLAG antibodies. They used anti-FLAG antibodies to detect the 3XFLAG-tagged proteins. Their data indicated (1) that the stability of SMN 6B was intermediate between that of FL-SMN and Δ7-SMN, (2) that SMN 6B retained its ability to interact with an FL-SMN counterpart protein, Gemin 2 and (3) that SMN 6B was less localized in the nucleus compared with the cytoplasm.

## Figures and Tables

**Figure 1:**
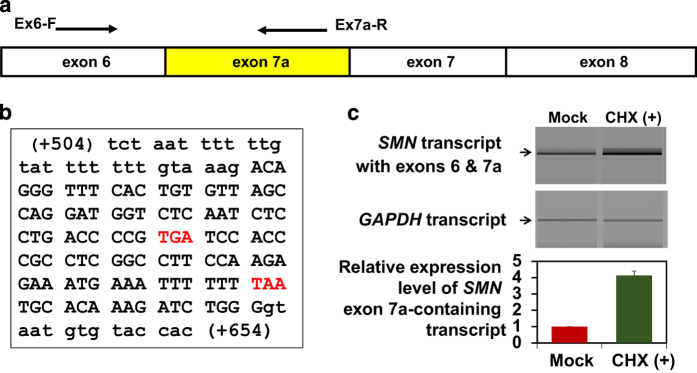
Detection of *SMN1/2* transcripts containing exons 6 and 7a and the effect of CHX on expression. (**a**) Scheme of the transcript and positions of RT–PCR primers. (**b**) Nucleotide sequence analysis of the PCR product from control fibroblasts. Uppercase letters indicate exon 7a nucleotides, and lowercase letters indicate nucleotides from flanking introns. Red letters indicate premature termination codons. (+504) and (+654) mean c.834+504 and c.834+654, which represent the locations of the first and last nucleotide in the table. (**c**) Semi-quantitative analysis using an Agilent 2100 Bioanalyzer. First, RT–PCR products for *SMN1/2* transcripts with exons 6 and 7a were separated by agarose gel electrophoresis. Then, *SMN1/2* transcripts with exons 6 and 7a (*SMN* exon 7a-containing transcript) were semi-quantified using a DNA 7500 LabChip Kit with an Agilent 2100 Bioanalyzer (Agilent Technologies, Inc., Santa Clara, CA, USA). In this study, ‘the relative expression level of the *SMN* exon 7a-containing transcript’ refers to the relative RT–PCR product ratio of the *SMN* exon 7a-containing transcript to the GAPDH transcript. The relative expression level of the exon 7a-containing *SMN* transcript in CHX-treated cells was significantly higher than that in non-treated control fibroblast cells (‘Mock’) (single-factor analysis of variance, *P*<0.001). The difference between them was almost fourfold.

**Figure 2:**
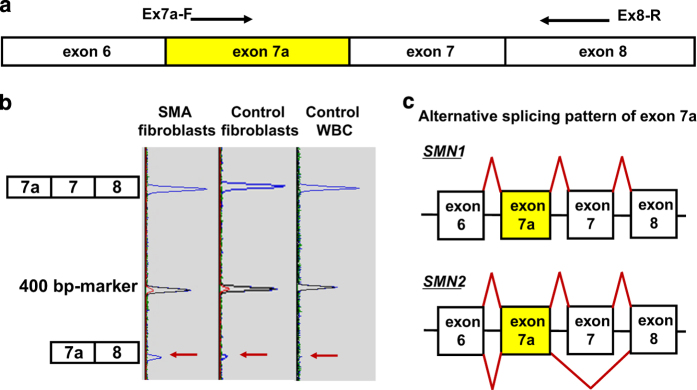
Alternatively spliced *SMN1/2* products containing exon 7a. (**a**) Scheme of the *SMN* transcript and positions of RT–PCR primers. (**b**) Fluorescent cDNA fragment analysis. To determine the ratio of alternatively spliced products, we performed a fluorescent cDNA fragment analysis using capillary electrophoresis. According to the manufacturer’s instructions, an aliquot of each purified sample was loaded with a DNA size marker (GeneScan-500 TAMRA; Applied Biosystems/Life Technologies Corporation, Carlsbad, CA, USA) and electrophoresed using the Genetic Analyzer (ABI PRISM 310; Applied Biosystems). Electrophoresis peaks were determined using GeneScan analysis software (Applied Biosystems). The transcript containing 'exon 6-exon 7a-exon 7-exon 8' was the major splicing product. The transcript with 'exon 6-exon 7a-exon 8' was not detected in the control white blood cells lacking *SMN2*. (**c**) Alternative splicing pattern of transcripts containing *SMN1/2* exon 7a. Boxes and horizontal lines represent exons and introns, respectively. Diagonal lines show alternative splicing patterns.

## References

[bib1] Nurputra DK, Lai PS, Harahap NI, Morikawa S, Yamamoto T, Nishimura N et al. Spinal muscular atrophy: from gene discovery to clinical trials. Ann Hum Genet 2013; 77: 435–463. 23879295 10.1111/ahg.12031

[bib2] Lefebvre S, Bürglen L, Reboullet S, Clermont O, Burlet P, Viollet L et al. Identification and characterization of a spinal muscular atrophy-determining gene. Cell 1995; 80: 155–165. 7813012 10.1016/0092-8674(95)90460-3

[bib3] Feldkötter M, Schwarzer V, Wirth R, Wienker TF, Wirth B. Quantitative analyses of SMN1 and SMN2 based on real-time light cycler PCR: fast and highly reliable carrier testing and prediction of severity of spinal muscular atrophy. Am J Hum Genet 2002; 70: 358–368. 11791208 10.1086/338627 PMC419987

[bib4] Yamamoto T, Sato H, Lai PS, Nurputra DK, Harahap NIF, Morikawa S et al. Intragenic mutations in *SMN1* may contribute more significantly to clinical severity than *SMN2* copy numbers in some spinal muscular atrophy (SMA) patients. Brain Dev 2014; 36: 914–920. 24359787 10.1016/j.braindev.2013.11.009

[bib5] Jodelka FM, Ebert AD, Duelli DM, Hastings ML. A feedback loop regulates splicing of the spinal muscular atrophy-modifying gene, SMN2. Hum Mol Genet 2010; 19: 4906–4917. 20884664 10.1093/hmg/ddq425 PMC2989896

[bib6] Lou K. Selectively splicing SMN2. SciBX 7(34)10.1038/scibx.2014.1001. Published online 4 September 2014. Available at http://www.nature.com/scibx/journal/v7/n34/full/scibx.2014.1001.html, accessed on 5 June 2016.

[bib7] Harahap NI, Nurputra DK, Rochmah M, Shima A, Morisada N, Takarada T et al. Salbutamol inhibits ubiquitin-mediated survival motor neuron protein degradation inspinal muscular atrophycells. Biochem Biophys Rep 2015; 4: 351–356. 10.1016/j.bbrep.2015.10.012 PMC5669398 29124224

[bib8] Brogna S, Wen J. Nonsense-mediated mRNA decay (NMD) mechanisms. Nat Struct Mol Biol 2009; 16: 107–113. 19190664 10.1038/nsmb.1550

[bib9] Carter MS, Doskow J, Morris P, Li S, Nhim RP, Sandstedt S et al. A regulatory mechanism that detects premature nonsense codons in T-cell receptor transcripts in vivo is reversed by protein synthesis inhibitors *in vitro*. J Biol Chem 1995; 48: 28995–29003. 10.1074/jbc.270.48.28995 7499432

[bib10] Matlin AJ, Clark F, Smith CW. Understanding alternative splicing: towards a cellular code. Nat Rev Mol Cell Biol 2005; 6: 386–398. 15956978 10.1038/nrm1645

[bib11] Seo JB, Singh NN, Ottesen EW, Lee BM, Singh RN. A novel human-specific splice isoform alters the critical C-terminus of Survival Motor Neuron protein. Sci Rep 2016; 6: 30778. 27481219 10.1038/srep30778 PMC4969610

